# Single-crystal X-ray diffraction dataset for 3,5-difluoro-2,6-bis(4-iodophenoxy)-4-phenoxypyridine

**DOI:** 10.1016/j.dib.2019.104956

**Published:** 2019-12-13

**Authors:** Andrew J. Peloquin, Khadijutal Kobra, Cynthia A. Corley, Colin D. McMillen, Timm A. Knoerzer, William T. Pennington, Scott T. Iacono

**Affiliations:** a Department of Chemistry, Clemson University, Clemson, SC, USA; b Department of Chemistry & Chemistry Research Center, Laboratories for Advanced Materials, United States Air Force Academy, Colorado Springs, CO, USA

**Keywords:** Pentafluoropyrinde, Perfluoropyridine, Halogen bonding, Solid state X-ray

## Abstract

The data in this article are related to the research article “Utilizing the Regioselectivity of Perfluoropyridine towards the Preparation of Phenyoxyacetylene Precursors for Partially Fluorinated Polymers of Diverse Architecture.”^1^ The X-ray structure analysis of 3,5-difluoro-2,6-bis(4-iodophenoxy)-4-phenoxypyridine has revealed an asymmetric unit containing two molecules, linked via both Type I and Type II C–I∙∙∙I–C halogen bonding interactions. The packing is further consolidated via Ar–H∙∙∙π interactions. This compound has been utilized for the synthesis of monomers for linear and network polymers.

**Table:** Specifications table

Subject	Organic Chemistry
Specific subject area	Solid-state interactions in 3,5-difluror-2,6-bis(4-iodophenoxy)-4-phenoxypyridine
Type of data	Tables of detailed X-ray crystal data and Cartesian coordinates Figures of X-ray crystal packing
How data were acquired	Single-crystal X-ray diffraction Instrument: Bruker D8 Venture diffractometer equipped with Mo Kα radiation and a Photon 100 detector Software: SHELXT 2014/5 and SHELXL 2016/6
Data format	X-ray (raw and analysed)
Parameters for data collection	Hemispheres of data were collected using strategies of scans about the omega and phi axes with frame widths of 0.5. Data collection, unit cell determination, data reduction, absorption correction, and scaling were performed using the Bruker Apex3 software suite: Apex3, AXScale and SAINT, version 2017.3-0; Bruker AXS Inc.: Madison, WI, 2017.
Data source location	Department of Chemistry and Chemistry Research Center, United States Air Force Academy, Colorado Springs, CO Department of Chemistry, Clemson university, Clemson, SC
Data accessibility	The Cambridge Crystallographic Data Centre no. CCDC 1947916 (http://www.ccdc.cam.ac.uk/conts/retrieving.html, email: deposit@ccdc.cam.ac.uk.).
Related research article	C.A. Corley, K. Kobra, A.J. Peloquin, K. Salmon, L. Gumireddy, T.A. Knoerzer, C.D. McMillen, W.T. Pennington, A.M. Schoffstall, S.T. Iacono. Utilizing the Regioselectivity of Perfluoropyridine towards the Preparation of Phenyoxyacetylene Precursors for Partially Fluorinated Polymers of Diverse Architecture, *J. Fluorine Chem.*, 2019, 228, IN PRESS, https://doi.org/10.1016/j.jfluchem.2019.109409.

**Table:** 

**Value of the Data** • The data points to the intermolecular halogen bonding in the solid state of the title compound. • Halogen bonding, being a specific and directional interaction, can be used to rationally design and modify solid state packing. • This compound has been demonstrated to be a versatile starting point for the synthesis of monomeric materials for high performance polymers.

## 1 Data

**Fig. 1:**
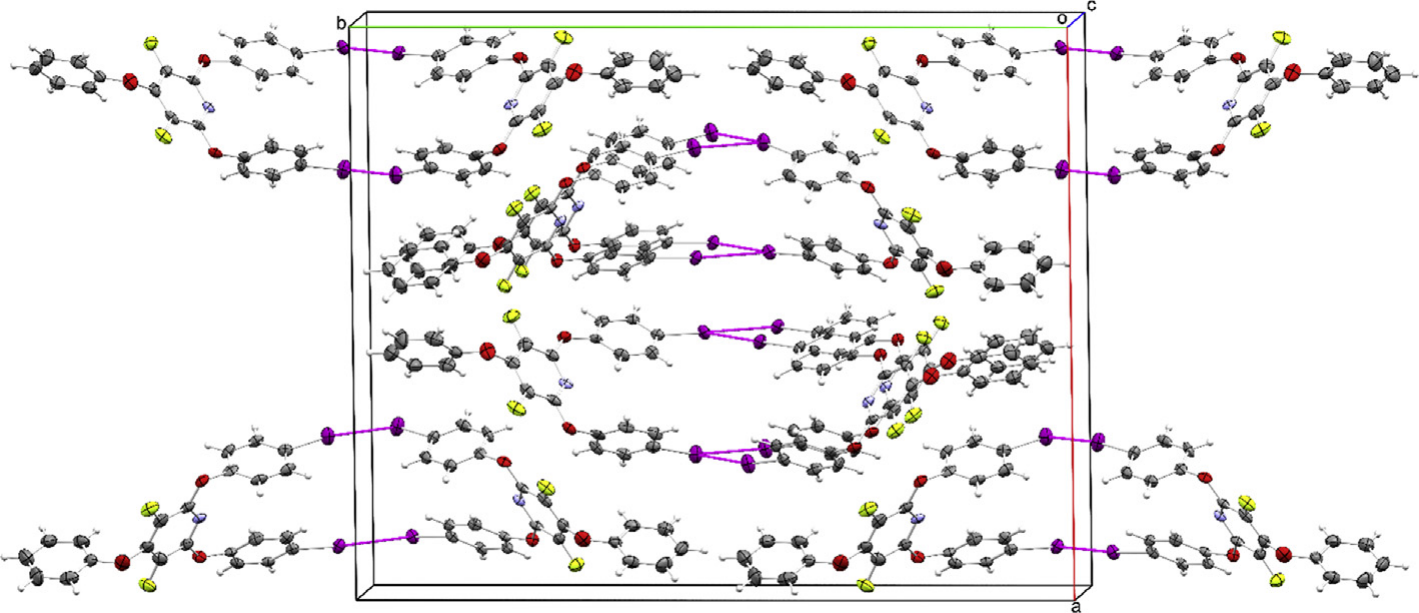
The crystal packing of D8_1390_PFPyPhI. Displacement ellipsoids are shown at the 50% probability level. Ar–I∙∙∙I interactions are shown as magenta dashed lines.

**Fig. 2:**
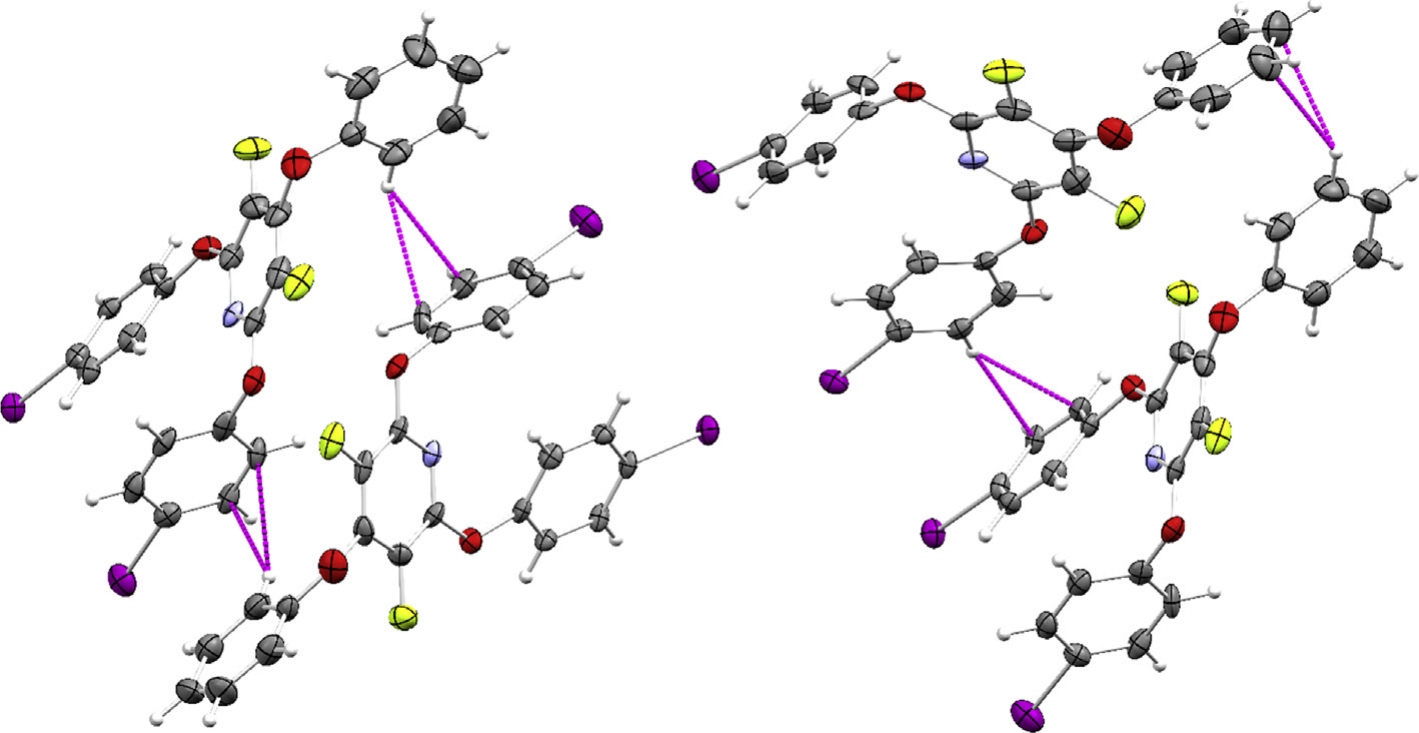
Ar–H∙∙∙π interactions in D8_1390_PFPyPhI (shown as magenta dashed lines). Displacement ellipsoids are shown at the 50% probability level.

The crystal packing of 3,5-difluoro-2,6-bis(4-iodophenoxy)-4-phenoxypyridine is shown, with C–I∙∙∙I–C Type I and Type II (Fig. 1) and Ar–H∙∙∙π (Fig. 2) interactions highlighted. What are also presented in the article are the detailed X-ray crystal data (Table 1, Table 2), atomic coordinates (Table 3), and bond lengths and angles (Table 4, Table 5, Table 6, Table 7).

**Table 1:** Sample and crystal data for D8_1390_PFPyPhI.

Identification code	D8_1390_PFPyPhI	
Chemical formula	C_23_H_13_F_2_I_2_NO_3_	
Formula weight	643.14 g/mol	
Temperature	100 (2) K	
Wavelength	0.71073 Å	
Crystal size	0.067 × 0.088 × 0.450 mm	
Crystal system	orthorhombic	
Space group	P n n 2	
Unit cell dimensions	a = 23.9615 (11) Å	α = 90°
	b = 30.1347 (13) Å	β = 90°
	c = 5.8713 (2) Å	γ = 90°
Volume	4239.5 (3) Å^3^	
Z	8	
Density (calculated)	2.015 g/cm^3^	
Absorption coefficient	3.010 mm^−1^	
F(000)	2448

**Table 2:** Data collection and structure refinement for D8_1390_PFPyPhI.

Theta range for data collection	2.64–25.50°	
Index ranges	−29 ≤ h<=28, −36 ≤ k<=36, −6 ≤ l<=7	
Reflections collected	34227	
Independent reflections	7300 [R (int) = 0.0568]	
Max. and min. transmission	1.0000 and 0.9225	
Structure solution technique	direct methods	
Structure solution program	SHELXT 2014/5 (Sheldrick, 2014)	
Refinement method	Full-matrix least-squares on F^2^	
Refinement program	SHELXL-2016/6 (Sheldrick, 2016)	
Function minimized	Σw (F_o_^2^ - F_c_^2^)^2^	
Data/restraints/parameters	7300/1/559	
Goodness-of-fit on F^2^	1.034	
Δ/σ_max_	0.001	
Final R indices	5749 data; I > 2σ(I)	R1 = 0.0392, wR2 = 0.0928
	all data	R1 = 0.0584, wR2 = 0.1029
Weighting scheme	w = 1/[σ^2^(F_o_^2^) + (0.0458P)^2^ + 10.9478P] where P = (F_o_^2^ + 2F_c_^2^)/3	
Absolute structure parameter	−0.009 (15)	
Largest diff. peak and hole	2.878 and −0.918 eÅ^−3^	
R.M.S. deviation from mean	0.137 eÅ^−3^

**Table 3:** Atomic coordinates and equivalent isotropic atomic displacement parameters (Å^2^) for D8_1390_PFPyPhI. U (eq) is defined as one third of the trace of the orthogonalized U_ij_ tensor.

	x/a	y/b	z/c	U (eq)
I1	0.57328 (3)	0.45589 (2)	0.92494 (12)	0.02996 (18)
I2	0.77564 (4)	0.46501 (2)	0.52911 (17)	0.0435 (2)
I3	0.56985 (3)	0.54613 (2)	0.42247 (12)	0.03046 (18)
I4	0.77216 (3)	0.54987 (2)	0.02299 (17)	0.0400 (2)
F1	0.6935 (3)	0.2367 (2)	0.6209 (10)	0.0373 (16)
F2	0.5454 (3)	0.2084 (2)	0.1314 (12)	0.0384 (16)
F3	0.6689 (3)	0.7766 (2)	0.1299 (11)	0.0443 (18)
F4	0.5219 (3)	0.7947 (2)	0.6569 (13)	0.0477 (19)
O1	0.5822 (3)	0.2758 (2)	0.3963 (13)	0.0259 (16)
O2	0.7308 (3)	0.3016 (2)	0.9043 (13)	0.0272 (16)
O3	0.6013 (3)	0.1890 (3)	0.7198 (14)	0.039 (2)
O4	0.5648 (3)	0.7278 (2)	0.9081 (14)	0.0294 (16)
O5	0.7107 (3)	0.7100 (2)	0.3974 (13)	0.0286 (16)
O6	0.5721 (3)	0.8189 (3)	0.2461 (15)	0.045 (2)
N1	0.6573 (3)	0.2874 (3)	0.1526 (15)	0.0232 (19)
N2	0.6388 (3)	0.7205 (3)	0.6532 (15)	0.0239 (19)
C1	0.5781 (4)	0.3963 (3)	0.7419 (18)	0.023 (2)
C2	0.5554 (4)	0.3582 (3)	0.8307 (17)	0.022 (2)
C3	0.5593 (4)	0.3186 (3)	0.7111 (18)	0.022 (2)
C4	0.5851 (4)	0.3171 (3)	0.502 (2)	0.022 (2)
C5	0.6080 (4)	0.3558 (3)	0.4080 (18)	0.023 (2)
C6	0.6045 (4)	0.3951 (3)	0.526 (2)	0.023 (2)
C7	0.7624 (4)	0.4101 (3)	0.3158 (18)	0.026 (2)
C8	0.7812 (4)	0.3691 (4)	0.3850 (19)	0.032 (3)
C9	0.7701 (5)	0.3323 (3)	0.2490 (19)	0.031 (3)
C10	0.7418 (4)	0.3386 (3)	0.045 (2)	0.025 (2)
C11	0.7222 (4)	0.3786 (3)	0.9779 (18)	0.029 (3)
C12	0.7332 (4)	0.4156 (3)	0.1121 (18)	0.030 (3)
C13	0.6117 (4)	0.2651 (3)	0.2045 (18)	0.024 (2)
C14	0.6839 (4)	0.2779 (3)	0.9639 (16)	0.023 (2)
C15	0.6665 (4)	0.2439 (4)	0.8199 (18)	0.027 (2)
C16	0.6202 (5)	0.2196 (3)	0.877 (2)	0.030 (3)
C17	0.5924 (4)	0.2306 (3)	0.073 (2)	0.027 (3)
C18	0.5939 (4)	0.1451 (4)	0.777 (2)	0.030 (3)
C19	0.6181 (4)	0.1259 (4)	0.966 (2)	0.041 (3)
C20	0.6110 (5)	0.0809 (4)	0.004 (3)	0.041 (3)
C21	0.5812 (5)	0.0545 (4)	0.848 (3)	0.052 (4)
C22	0.5580 (5)	0.0749 (4)	0.661 (3)	0.050 (4)
C23	0.5641 (5)	0.1201 (4)	0.621 (2)	0.040 (3)
C24	0.5695 (4)	0.6063 (3)	0.2409 (18)	0.024 (2)
C25	0.5450 (4)	0.6429 (3)	0.3354 (18)	0.024 (2)
C26	0.5452 (4)	0.6831 (3)	0.2201 (18)	0.025 (2)
C27	0.5706 (4)	0.6863 (3)	0.010 (2)	0.023 (2)
C28	0.5963 (4)	0.6486 (3)	0.913 (2)	0.030 (2)
C29	0.5950 (4)	0.6091 (3)	0.030 (2)	0.028 (2)
C30	0.7535 (4)	0.6037 (4)	0.8061 (19)	0.029 (3)
C31	0.7249 (4)	0.5958 (4)	0.6057 (19)	0.034 (3)
C32	0.7106 (4)	0.6324 (3)	0.4692 (18)	0.030 (3)
C33	0.7251 (4)	0.6739 (3)	0.535 (2)	0.029 (2)
C34	0.7532 (5)	0.6814 (4)	0.7389 (19)	0.029 (3)
C35	0.7678 (4)	0.6461 (3)	0.8745 (18)	0.028 (2)
C36	0.5915 (4)	0.7407 (4)	0.7151 (19)	0.028 (2)
C37	0.6631 (4)	0.7324 (3)	0.4639 (17)	0.028 (3)
C38	0.6432 (5)	0.7665 (4)	0.3287 (19)	0.032 (3)
C39	0.5953 (4)	0.7879 (4)	0.393 (2)	0.035 (3)
C40	0.5696 (5)	0.7749 (4)	0.589 (2)	0.036 (3)
C41	0.5747 (4)	0.8642 (4)	0.295 (2)	0.033 (3)
C42	0.6020 (5)	0.8813 (4)	0.480 (2)	0.043 (3)
C43	0.6030 (5)	0.9271 (4)	0.507 (3)	0.049 (3)
C44	0.5775 (6)	0.9553 (5)	0.358 (3)	0.059 (5)
C45	0.5498 (6)	0.9366 (4)	0.168 (3)	0.059 (4)
C46	0.5475 (5)	0.8905 (4)	0.137 (2)	0.047 (3)

**Table 4:** Bond lengths (Å) for D8_1390_PFPyPhI.

I1–C1	2.095 (9)	I1–C2	3.026 (10)
I1–C6	3.065 (11)	I1–I3	3.9919 (9)
I2–C7	2.099 (10)	I2–C8	3.015 (11)
I2–C12	3.041 (11)	I2–I4	3.8673 (12)
I3–C24	2.104 (10)	I3–C25	3.019 (10)
I3–C29	3.046 (12)	I4–C30	2.109 (11)
I4–C35	3.031 (11)	I4–C31	3.033 (12)
F1–C15	1.353 (11)	F2–C17	1.356 (12)
F3–C38	1.354 (13)	F4–C40	1.351 (13)
O1–C13	1.369 (12)	O1–C4	1.393 (11)
O2–C14	1.377 (12)	O2–C10	1.413 (13)
O3–C18	1.377 (13)	O3–C16	1.379 (13)
O4–C36	1.358 (13)	O4–C27	1.393 (12)
O5–C37	1.381 (13)	O5–C33	1.400 (14)
O6–C39	1.388 (13)	O6–C41	1.394 (14)
N1–C14	1.310 (13)	N1–C13	1.319 (13)
N2–C37	1.305 (13)	N2–C36	1.337 (14)
C1–C2	1.374 (14)	C1–C6	1.416 (16)
C2–C3	1.387 (14)	C2–H2	0.95
C3–C4	1.375 (15)	C3–H3	0.95
C4–C5	1.402 (14)	C5–C6	1.375 (14)
C5–H5	0.95	C6–H6	0.95
C7–C8	1.377 (14)	C7–C12	1.396 (15)
C8–C9	1.390 (16)	C8–H8	0.95
C9–C10	1.388 (17)	C9–H9	0.95
C10–C11	1.355 (13)	C11–C12	1.389 (14)
C11–H11	0.95	C12–H12	0.95
C13–C17	1.372 (14)	C14–C15	1.392 (14)
C15–C16	1.372 (16)	C16–C17	1.374 (15)
C18–C19	1.379 (16)	C18–C23	1.384 (16)
C19–C20	1.383 (15)	C19–H19	0.95
C20–C21	1.406 (19)	C20–H20	0.95
C21–C22	1.38 (2)	C21–H21	0.95
C22–C23	1.388 (17)	C22–H22	0.95
C23–H23	0.95	C24–C25	1.365 (14)
C24–C29	1.385 (16)	C25–C26	1.388 (14)
C25–H25	0.95	C26–C27	1.377 (16)
C26–H26	0.95	C27–C28	1.413 (14)
C28–C29	1.372 (14)	C28–H28	0.95
C29–H29	0.95	C30–C31	1.382 (15)
C30–C35	1.385 (15)	C31–C32	1.407 (15)
C31–H31	0.95	C32–C33	1.353 (14)
C32–H32	0.95	C33–C34	1.392 (18)
C34–C35	1.374 (15)	C34–H34	0.95
C35–H35	0.95	C36–C40	1.372 (15)
C37–C38	1.384 (15)	C38–C39	1.367 (16)
C39–C40	1.366 (16)	C41–C42	1.370 (17)
C41–C46	1.384 (16)	C42–C43	1.388 (16)
C42–H42	0.95	C43–C44	1.36 (2)
C43–H43	0.95	C44–C45	1.42 (2)
C44–H44	0.95	C45–C46	1.404 (17)
C45–H45	0.95	C46–H46	0.95

**Table 5:** Bond angles (°) for D8_1390_PFPyPhI.

C1–I1–C2	23.2 (4)	C1–I1–C6	23.5 (3)
C2–I1–C6	46.6 (3)	C1–I1–I3	163.8 (3)
C2–I1–I3	142.52 (19)	C6–I1–I3	166.18 (17)
C7–I2–C8	23.6 (4)	C7–I2–C12	23.5 (4)
C8–I2–C12	47.1 (3)	C7–I2–I4	164.9 (3)
C8–I2–I4	147.7 (2)	C12–I2–I4	156.99 (19)
C24–I3–C25	23.2 (4)	C24–I3–C29	23.1 (4)
C25–I3–C29	46.3 (3)	C24–I3–I1	102.5 (3)
C25–I3–I1	122.5 (2)	C29–I3–I1	82.3 (2)
C30–I4–C35	23.6 (4)	C30–I4–C31	23.4 (4)
C35–I4–C31	47.0 (3)	C30–I4–I2	93.4 (3)
C35–I4–I2	114.70 (19)	C31–I4–I2	72.8 (2)
C13–O1–C4	123.5 (8)	C14–O2–C10	114.4 (7)
C18–O3–C16	121.5 (9)	C36–O4–C27	124.7 (8)
C37–O5–C33	114.8 (8)	C39–O6–C41	120.9 (9)
C14–N1–C13	119.1 (9)	C37–N2–C36	119.0 (10)
C2–C1–C6	119.6 (9)	C2–C1–I1	120.0 (8)
C6–C1–I1	120.4 (7)	C1–C2–C3	120.0 (10)
C1–C2–I1	36.9 (5)	C3–C2–I1	156.8 (7)
C1–C2–H2	120.0	C3–C2–H2	120.0
I1–C2–H2	83.2	C4–C3–C2	120.7 (9)
C4–C3–H3	119.7	C2–C3–H3	119.7
C3–C4–O1	113.8 (9)	C3–C4–C5	120.0 (9)
O1–C4–C5	126.0 (10)	C6–C5–C4	119.5 (10)
C6–C5–H5	120.2	C4–C5–H5	120.2
C5–C6–C1	120.1 (9)	C5–C6–I1	156.2 (8)
C1–C6–I1	36.1 (5)	C5–C6–H6	119.9
C1–C6–H6	119.9	I1–C6–H6	83.8
C8–C7–C12	121.5 (10)	C8–C7–I2	118.9 (8)
C12–C7–I2	119.6 (8)	C7–C8–C9	119.0 (10)
C7–C8–I2	37.6 (6)	C9–C8–I2	156.4 (8)
C7–C8–H8	120.5	C9–C8–H8	120.5
I2–C8–H8	83.0	C10–C9–C8	118.7 (9)
C10–C9–H9	120.7	C8–C9–H9	120.7
C11–C10–C9	122.8 (11)	C11–C10–O2	117.8 (11)
C9–C10–O2	119.3 (9)	C10–C11–C12	118.8 (10)
C10–C11–H11	120.6	C12–C11–H11	120.6
C11–C12–C7	119.1 (9)	C11–C12–I2	155.9 (7)
C7–C12–I2	36.9 (5)	C11–C12–H12	120.4
C7–C12–H12	120.4	I2–C12–H12	83.6
N1–C13–O1	119.8 (9)	N1–C13–C17	122.4 (10)
O1–C13–C17	117.8 (9)	N1–C14–O2	119.9 (9)
N1–C14–C15	122.0 (10)	O2–C14–C15	118.1 (9)
F1–C15–C16	120.6 (10)	F1–C15–C14	120.0 (10)
C16–C15–C14	119.2 (10)	C15–C16–C17	117.7 (10)
C15–C16–O3	117.5 (11)	C17–C16–O3	124.4 (10)
F2–C17–C13	120.9 (10)	F2–C17–C16	119.6 (9)
C13–C17–C16	119.5 (10)	O3–C18–C19	123.0 (10)
O3–C18–C23	115.3 (11)	C19–C18–C23	121.5 (11)
C18–C19–C20	119.1 (12)	C18–C19–H19	120.4
C20–C19–H19	120.4	C19–C20–C21	120.9 (13)
C19–C20–H20	119.5	C21–C20–H20	119.5
C22–C21–C20	117.9 (12)	C22–C21–H21	121.0
C20–C21–H21	121.0	C21–C22–C23	122.1 (12)
C21–C22–H22	118.9	C23–C22–H22	118.9
C18–C23–C22	118.3 (12)	C18–C23–H23	120.8
C22–C23–H23	120.8	C25–C24–C29	120.3 (10)
C25–C24–I3	119.4 (8)	C29–C24–I3	120.2 (7)
C24–C25–C26	120.3 (10)	C24–C25–I3	37.4 (5)
C26–C25–I3	157.7 (8)	C24–C25–H25	119.8
C26–C25–H25	119.8	I3–C25–H25	82.5
C27–C26–C25	119.9 (10)	C27–C26–H26	120.0
C25–C26–H26	120.0	C26–C27–O4	113.9 (9)
C26–C27–C28	119.7 (10)	O4–C27–C28	126.3 (10)
C29–C28–C27	119.2 (10)	C29–C28–H28	120.4
C27–C28–H28	120.4	C28–C29–C24	120.5 (10)
C28–C29–I3	157.1 (8)	C24–C29–I3	36.6 (5)
C28–C29–H29	119.7	C24–C29–H29	119.7
I3–C29–H29	83.1	C31–C30–C35	121.9 (11)
C31–C30–I4	119.1 (8)	C35–C30–I4	118.8 (8)
C30–C31–C32	118.1 (10)	C30–C31–I4	37.4 (6)
C32–C31–I4	155.4 (7)	C30–C31–H31	121.0
C32–C31–H31	121.0	I4–C31–H31	83.6
C33–C32–C31	120.0 (10)	C33–C32–H32	120.0
C31–C32–H32	120.0	C32–C33–C34	121.4 (11)
C32–C33–O5	119.3 (11)	C34–C33–O5	119.3 (9)
C35–C34–C33	119.7 (10)	C35–C34–H34	120.2
C33–C34–H34	120.2	C34–C35–C30	119.0 (10)
C34–C35–I4	156.4 (8)	C30–C35–I4	37.6 (6)
C34–C35–H35	120.5	C30–C35–H35	120.5
I4–C35–H35	83.0	N2–C36–O4	119.8 (9)
N2–C36–C40	121.3 (10)	O4–C36–C40	119.0 (10)
N2–C37–O5	118.4 (9)	N2–C37–C38	122.6 (11)
O5–C37–C38	119.1 (9)	F3–C38–C39	120.8 (10)
F3–C38–C37	120.3 (11)	C39–C38–C37	118.8 (10)
C40–C39–C38	118.4 (11)	C40–C39–O6	122.5 (11)
C38–C39–O6	118.9 (11)	F4–C40–C39	120.2 (11)
F4–C40–C36	119.9 (11)	C39–C40–C36	119.9 (11)
C42–C41–C46	122.7 (12)	C42–C41–O6	123.7 (10)
C46–C41–O6	113.6 (11)	C41–C42–C43	118.1 (12)
C41–C42–H42	120.9	C43–C42–H42	120.9
C44–C43–C42	122.8 (15)	C44–C43–H43	118.6
C42–C43–H43	118.6	C43–C44–C45	117.9 (13)
C43–C44–H44	121.0	C45–C44–H44	121.0
C46–C45–C44	120.8 (12)	C46–C45–H45	119.6
C44–C45–H45	119.6	C41–C46–C45	117.7 (13)
C41–C46–H46	121.2	C45–C46–H46	121.2

**Table 6:** Anisotropic atomic displacement parameters (Å^2^) for D8_1390_PFPyPhI. The anisotropic atomic displacement factor exponent takes the form: 2π^2^ [h^2^ a*^2^ U_11_ + ... + 2 h k a* b* U_12_].

	U_11_	U_22_	U_33_	U_23_	U_13_	U_12_
I1	0.0390 (4)	0.0297 (3)	0.0212 (4)	−0.0053 (4)	0.0019 (4)	0.0059 (3)
I2	0.0665 (5)	0.0345 (4)	0.0295 (4)	0.0000 (5)	−0.0012 (5)	−0.0088 (4)
I3	0.0388 (4)	0.0310 (3)	0.0216 (4)	0.0018 (4)	0.0017 (4)	−0.0062 (3)
I4	0.0577 (5)	0.0340 (4)	0.0282 (4)	−0.0037 (5)	0.0000 (4)	0.0005 (3)
F1	0.044 (4)	0.052 (4)	0.016 (3)	−0.006 (3)	−0.001 (3)	0.020 (3)
F2	0.036 (4)	0.040 (4)	0.039 (4)	−0.012 (3)	0.004 (3)	−0.007 (3)
F3	0.041 (4)	0.067 (5)	0.025 (4)	0.017 (3)	−0.005 (3)	−0.020 (3)
F4	0.036 (4)	0.059 (4)	0.048 (5)	0.013 (4)	−0.003 (4)	0.016 (3)
O1	0.032 (4)	0.027 (3)	0.019 (4)	−0.005 (3)	0.005 (3)	0.001 (3)
O2	0.022 (4)	0.037 (4)	0.022 (4)	−0.002 (4)	0.007 (4)	0.007 (3)
O3	0.049 (5)	0.045 (5)	0.024 (5)	−0.011 (4)	−0.011 (4)	0.006 (4)
O4	0.026 (4)	0.038 (4)	0.025 (4)	0.009 (4)	0.007 (4)	0.004 (3)
O5	0.029 (4)	0.036 (4)	0.021 (4)	0.007 (3)	0.002 (3)	−0.012 (3)
O6	0.053 (6)	0.047 (5)	0.035 (5)	0.010 (4)	−0.023 (4)	−0.006 (4)
N1	0.025 (5)	0.027 (5)	0.018 (5)	−0.002 (4)	0.000 (4)	0.008 (4)
N2	0.021 (5)	0.035 (5)	0.016 (5)	0.001 (4)	−0.004 (4)	−0.009 (4)
C1	0.027 (6)	0.027 (5)	0.015 (6)	−0.005 (4)	0.000 (4)	0.010 (4)
C2	0.028 (5)	0.028 (5)	0.010 (5)	0.004 (4)	0.002 (4)	0.011 (4)
C3	0.022 (5)	0.026 (5)	0.019 (6)	0.005 (4)	0.004 (4)	0.003 (4)
C4	0.020 (5)	0.027 (5)	0.020 (6)	−0.002 (5)	0.002 (4)	0.008 (4)
C5	0.028 (5)	0.033 (5)	0.009 (5)	−0.001 (5)	0.001 (5)	0.003 (4)
C6	0.023 (5)	0.026 (5)	0.021 (5)	0.005 (6)	0.003 (5)	0.000 (4)
C7	0.032 (6)	0.026 (6)	0.022 (6)	−0.001 (4)	0.003 (5)	−0.005 (5)
C8	0.029 (6)	0.047 (6)	0.020 (7)	0.009 (5)	0.000 (5)	0.010 (5)
C9	0.042 (7)	0.025 (6)	0.026 (6)	0.006 (5)	0.007 (5)	0.018 (5)
C10	0.024 (5)	0.030 (5)	0.023 (6)	0.008 (6)	0.011 (5)	0.000 (4)
C11	0.028 (6)	0.035 (6)	0.022 (8)	0.008 (4)	0.001 (4)	0.002 (4)
C12	0.029 (6)	0.030 (6)	0.031 (7)	0.011 (4)	0.004 (5)	0.004 (5)
C13	0.019 (5)	0.034 (6)	0.019 (6)	−0.003 (4)	0.002 (4)	0.008 (5)
C14	0.022 (5)	0.031 (5)	0.015 (6)	0.001 (4)	0.002 (4)	0.012 (4)
C15	0.036 (6)	0.035 (6)	0.010 (5)	−0.002 (4)	0.000 (5)	0.020 (5)
C16	0.035 (6)	0.026 (5)	0.029 (7)	−0.010 (5)	−0.008 (5)	0.007 (5)
C17	0.025 (6)	0.027 (5)	0.029 (8)	−0.003 (5)	−0.004 (5)	−0.001 (4)
C18	0.026 (6)	0.030 (6)	0.034 (7)	−0.009 (5)	−0.003 (5)	0.005 (5)
C19	0.026 (6)	0.047 (7)	0.049 (10)	−0.009 (6)	−0.001 (5)	0.003 (5)
C20	0.035 (6)	0.046 (7)	0.043 (8)	0.000 (7)	−0.002 (6)	0.004 (5)
C21	0.039 (7)	0.034 (7)	0.084 (13)	−0.013 (7)	0.010 (7)	−0.010 (6)
C22	0.047 (8)	0.047 (8)	0.056 (10)	−0.023 (7)	−0.006 (7)	−0.010 (6)
C23	0.034 (7)	0.051 (8)	0.034 (8)	−0.011 (6)	−0.008 (5)	0.007 (6)
C24	0.024 (6)	0.032 (6)	0.016 (6)	0.003 (4)	−0.001 (4)	−0.003 (4)
C25	0.019 (5)	0.035 (6)	0.018 (6)	−0.003 (4)	−0.002 (4)	−0.002 (4)
C26	0.023 (5)	0.035 (6)	0.017 (6)	−0.001 (4)	−0.005 (4)	0.003 (5)
C27	0.015 (5)	0.031 (5)	0.024 (6)	−0.002 (5)	0.004 (5)	−0.003 (4)
C28	0.032 (6)	0.044 (6)	0.015 (6)	0.005 (5)	0.004 (6)	−0.007 (5)
C29	0.033 (6)	0.033 (5)	0.019 (5)	−0.002 (6)	−0.004 (6)	−0.005 (4)
C30	0.027 (6)	0.039 (6)	0.021 (6)	−0.001 (5)	0.003 (5)	0.000 (5)
C31	0.033 (6)	0.040 (6)	0.030 (7)	−0.016 (5)	0.002 (5)	−0.011 (5)
C32	0.023 (5)	0.047 (6)	0.020 (7)	−0.013 (5)	−0.002 (4)	−0.009 (5)
C33	0.023 (5)	0.042 (6)	0.022 (5)	−0.005 (7)	0.014 (5)	−0.011 (4)
C34	0.035 (6)	0.032 (6)	0.021 (6)	0.000 (5)	0.003 (5)	−0.016 (5)
C35	0.028 (6)	0.040 (6)	0.016 (6)	−0.009 (5)	0.002 (4)	−0.011 (5)
C36	0.026 (6)	0.037 (6)	0.021 (6)	0.011 (5)	−0.003 (5)	−0.008 (5)
C37	0.026 (6)	0.040 (6)	0.018 (7)	0.002 (4)	−0.002 (4)	−0.014 (5)
C38	0.039 (7)	0.043 (7)	0.016 (6)	0.011 (5)	−0.008 (5)	−0.017 (5)
C39	0.028 (6)	0.045 (7)	0.032 (8)	0.019 (6)	−0.013 (6)	−0.009 (5)
C40	0.036 (7)	0.042 (7)	0.029 (8)	0.003 (5)	−0.008 (5)	0.002 (5)
C41	0.026 (6)	0.038 (6)	0.034 (8)	0.013 (5)	−0.001 (5)	−0.003 (5)
C42	0.035 (7)	0.057 (7)	0.038 (9)	0.020 (6)	−0.011 (5)	−0.001 (5)
C43	0.030 (6)	0.050 (7)	0.067 (10)	−0.008 (8)	−0.002 (7)	−0.003 (5)
C44	0.052 (9)	0.048 (8)	0.078 (14)	0.013 (7)	−0.006 (8)	0.011 (7)
C45	0.070 (10)	0.041 (8)	0.065 (11)	0.023 (7)	−0.012 (8)	0.002 (7)
C46	0.037 (7)	0.074 (10)	0.032 (7)	0.017 (7)	−0.006 (6)	−0.011 (7)

**Table 7:** Hydrogen atomic coordinates and isotropic atomic displacement parameters (Å^2^) for D8_1390_PFPyPhI.

	x/a	y/b	z/c	U (eq)
H2	0.5370	0.3590	0.9741	0.027
H3	0.5439	0.2923	0.7741	0.027
H5	0.6258	0.3549	0.2635	0.028
H6	0.6198	0.4214	0.4632	0.028
H8	0.8013	0.3659	0.5235	0.039
H9	0.7818	0.3035	0.2946	0.037
H11	0.7012	0.3814	−0.1587	0.034
H12	0.7210	0.4442	0.0657	0.035
H19	0.6394	0.1433	0.0689	0.049
H20	0.6264	0.0678	0.1363	0.05
H21	0.5772	0.0235	−0.1283	0.063
H22	0.5371	0.0576	−0.4437	0.06
H23	0.5483	0.1335	−0.5106	0.048
H25	0.5278	0.6408	1.4807	0.029
H26	0.5279	0.7084	1.2859	0.03
H28	0.6143	0.6505	0.7695	0.036
H29	0.6117	0.5835	0.9649	0.034
H31	0.7152	0.5664	0.5615	0.041
H32	0.6909	0.6281	0.3307	0.036
H34	0.7623	0.7108	0.7840	0.035
H35	0.7875	0.6508	1.0129	0.034
H42	0.6197	0.8624	0.5878	0.052
H43	0.6222	0.9393	0.6334	0.059
H44	0.5783	0.9865	0.3813	0.071
H45	0.5325	0.9556	0.0591	0.071
H46	0.5280	0.8777	0.0123	0.057

## 2 Experimental design, materials, and methods

Compound **3** was successfully crystallized through slow evaporation of an ethanol solution. Single crystal X-ray diffraction data were obtained at 100 K using a Bruker D8 Venture diffractometer equipped with Mo Kα radiation and a Photon 100 detector. Structure solution and refinement were performed using SHELXT 2014/5 and SHELXL 2016/6. All non-hydrogen atoms were refined anisotropically, while hydrogen atoms were placed geometrically and refined using riding models.

The structure was test-refined in Ccc2, with that model consisting of one unique molecule having highly correlated thermal ellipsoids, some of which should be split, suggesting disorder or lower symmetry than Ccc2. Furthermore, there are 6631 systematic absence violations (many for hkl, h + k = odd) in the Ccc2 model, versus 0 in the Pnn2 model. Thus, the current model in Pnn2 would seem to better fit the true symmetry of the system, having two unique molecules in the asymmetric unit with well-behaved ADPs.

CCDC contains the supplementary crystallographic data for this paper. The CCDC reference number is 1947916. These data can be viewed free of charge via http://www.ccdc.cam.ac.uk/cont/retrieving.html or from the CCDC, 12 Union Road, Cambridge CB21EZ, UK. Fax: +44 1223 336033. E-mail: deposit@ccdc.cam.ac.uk.

